# Ultrasound Evaluation and Surgical Excision of a Fabella Causing Peroneal Neuropathy in a Track Athlete

**DOI:** 10.1155/2018/2371947

**Published:** 2018-12-30

**Authors:** Kevin M. Dale, Samuel B. Boggess, Blake Boggess, Claude T. Moorman

**Affiliations:** ^1^Division of Pediatric Orthopaedics, Department of Orthopaedic Surgery, Monroe Carell Jr. Children's Hospital at Vanderbilt, Vanderbilt University School of Medicine, Nashville, TN, USA; ^2^Department of Exercise Sciences, Brigham Young University, Provo, UT, USA; ^3^Duke Sports Medicine Team Physician, Duke University School of Medicine, Durham, NC, USA; ^4^Department of Orthopedics, Carolinas Medical Center, Charlotte, NC, USA

## Abstract

**Background:**

There are multiple causes of posterior knee pain and radicular symptoms. A symptomatic fabella is a rare cause but should be considered in the differential diagnosis.

**Purpose:**

Physicians should consider a symptomatic fabella as a diagnosis when common treatments for posterior knee pain have not alleviated the symptoms.

**Study Design:**

Case report.

**Methods:**

Review of clinical documentations of an orthopedist, physiatrist, physical therapist, 2 primary care sports medicine physicians, and the surgical report of an orthopedist.

**Results:**

It took time and resources including several referrals and imaging modalities to make a final diagnosis.

**Conclusion:**

Symptomatic fabellae are an uncommon finding but should be considered in the differential diagnosis with an athlete with posterior knee pain.

**Clinical Relevance:**

Considerable time and resources were used to ultimately diagnose and treat a NCAA Division 1 athlete. Surgical excision was required of a sesamoid bone that is present in 30% of individuals.

## 1. Introduction

The fabella is a sesamoid bone that is present posterior to the knee in up to 30% of people and may be bilateral 80% of these individuals [1]. When the fabella is present, it is located in the tendon of the lateral head of the gastrocnemius behind the lateral posterior condyle of the knee up to 87-97% of the time compared to the medial head [2, 3]. The fabella has been shown to have a higher incidence in patients with Asian descent [3]. In its role of stabilizing the soft tissue structures of the posterolateral knee, the fabella seems to serve as suspension for the ligaments evolving from its base [4].

Originally described in 1929, “fabella syndrome” has been described as a posterior lateral knee pain in the presence of a fabella [5]. The pain occurs when the knee is brought from flexion to extension causing tensioning of the gastrocnemius tendon with irritation of the fabella on the posterior lateral femoral condyle. Good results have been found with the excision of the fabella in symptomatic patients [6–8].

Although less common, the fabella has also been known to cause peroneal neuropathy [9, 10]. Originally described in 1963 by Tabira et al. [11], case reports of the fabella causing symptoms of the peroneal nerve were reported mostly in the Japanese literature in the 1960's and 1970's [12–16]. Recently, there has been a renewed interest in compression neuropathy caused by the fabella [9, 17].

Patients will often present after a long period of diagnostic workup before the diagnosis of peroneal neuropathy caused by the fabella is reached [18]. Since the diagnosis is rare, it is often made as a diagnosis of exclusion. We would like to present the dynamic ultrasound evaluation along with a surgical management for treatment of peroneal neuropathy caused by the fabella.

## 2. Case Description

A 20-year-old male collegiate track athlete presented with 8 months of right posterolateral leg pain and paresthesia. The symptoms had begun shortly after starting to use a new pair of orthotics during his season. The symptoms were initially described as a sharp pain, present usually only with activity, rarely symptomatic at rest, and notably worse with going up stairs or from sitting to standing. He was initially treated for shin splints, and after a period of rest, placement in a walking boot, and physical therapy, the symptoms did improve slightly. He continued to have persistent tingling and intermittent shooting pains with long runs, sprints, playing basketball, and with lifting, most notably with squats. Physical exam was remarkable for numbness around the right fibular head with percussion or after exercise.

**Figure 1:**
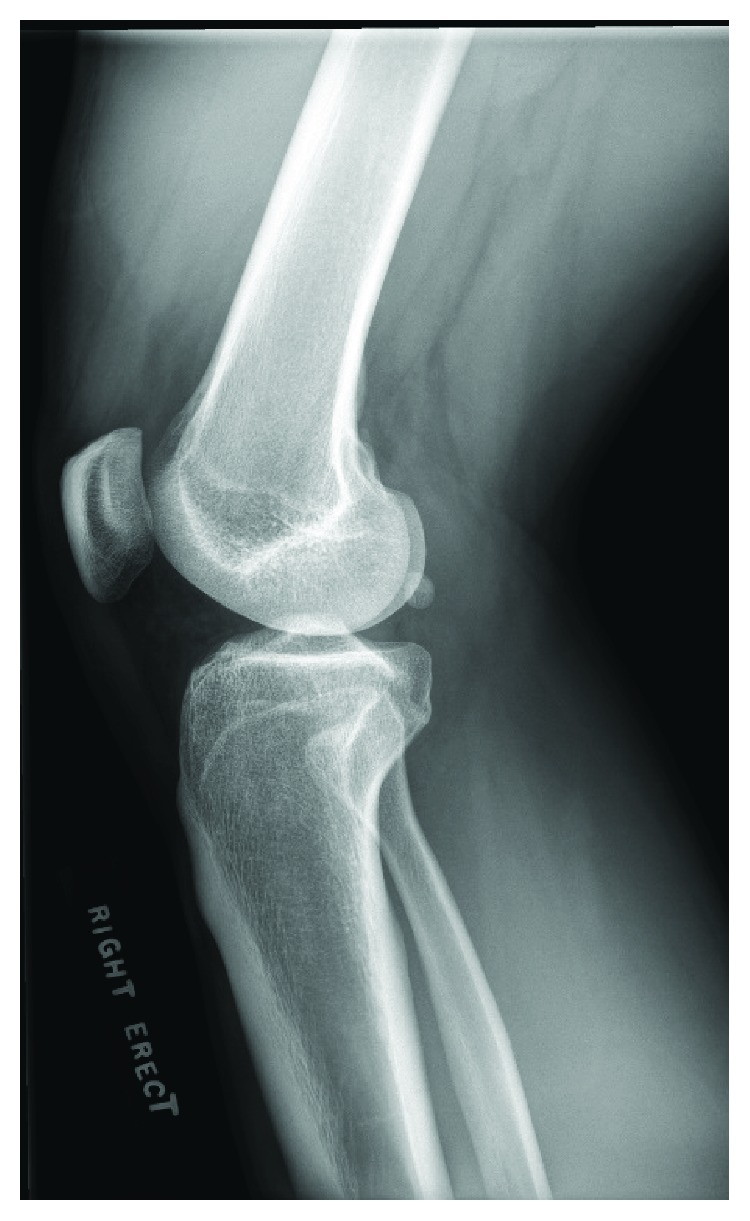
X-ray showing the fabella in a normal posterior location on the lateral X-ray.

**Figure 2:**
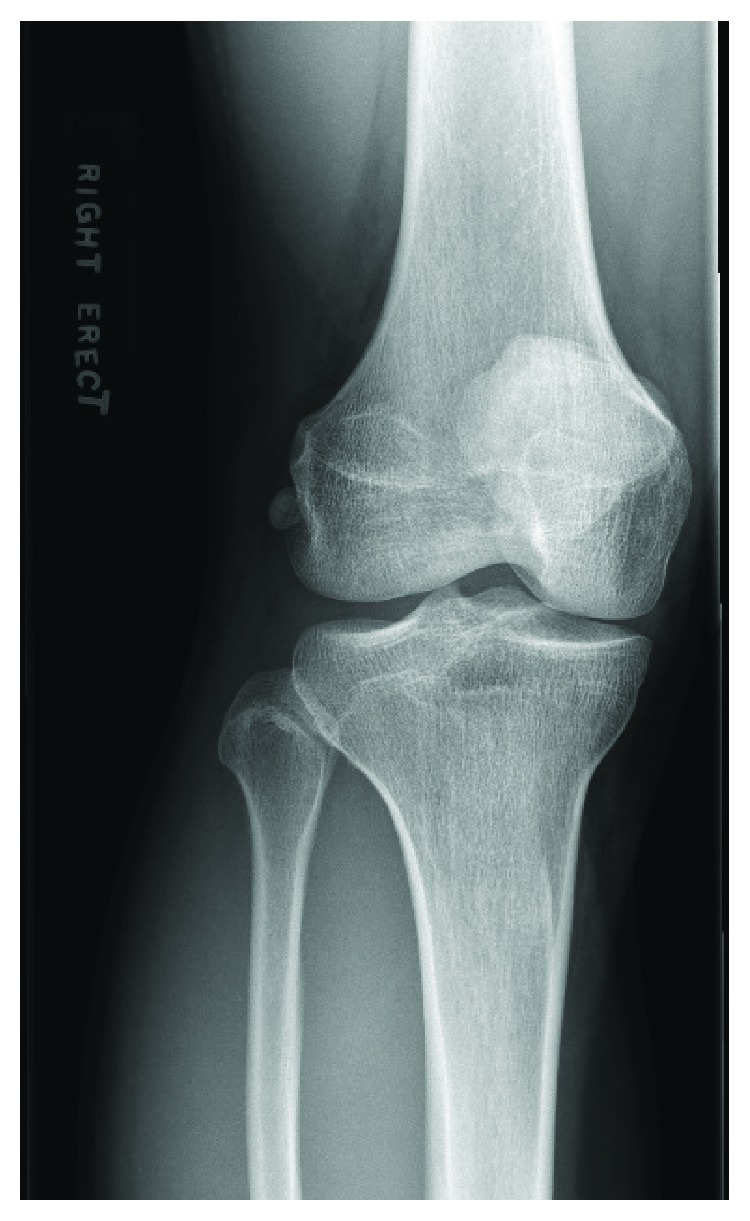
Fabella located lateral out from behind the lateral femur on the AP X-ray.

He was referred to orthopedics where X-rays of the knee were normal (Figures 1 and 2). There was concern for a possible lumbar radiculopathy, so imaging of his lumber spine was obtained. He was started on a course of prednisone and was referred to a physical medicine and rehabilitation specialist for further evaluation. There, his symptoms were localized around his fibular head and peroneal nerve, with no spinal involvement. He was sent to neurology for peripheral nerve testing. He underwent EMG and had an ultrasound of his peroneal nerve, which did show bilateral nerve enlargement, but with normal conduction. He was then referred to a primary care sports medicine specialist that performs compartment syndrome testing. These tests were mildly positive, but his symptoms were felt to be somewhat inconsistent with that diagnosis. The athlete was then sent to another primary care sports medicine physician that specializes in musculoskeletal ultrasound so that a dynamic evaluation of the posterior knee could be performed.

### 2.1. Ultrasound

**Figure 3:**
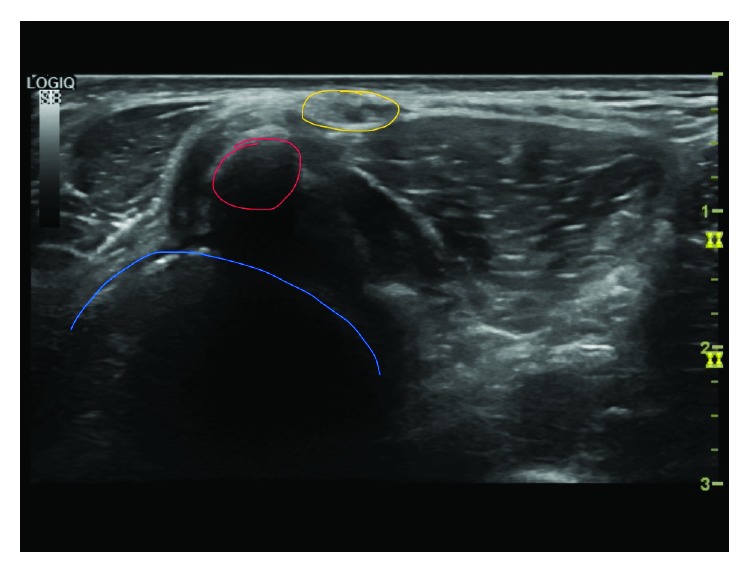
Axial ultrasound of the posterior lateral knee (blue: femoral condyle, red: fabella causing acoustic shadowing, and gold: peroneal nerve).

The dynamic ultrasound evaluation was performed using a GE S8 ultrasound machine with a 6-15 MHz transducer (General Electric, Chicago, Illinois, USA). The dynamic ultrasound showed a moderate-sized fabella in the posterior knee adjacent to the peroneal nerve, with a dynamic compression of the nerve on knee flexion and nerve enlargement distally (see Figure 3 and ultrasound videos 1 and 2). The patient underwent peroneal nerve hydrodissection with corticosteroid injection 8cc of 1% lidocaine and 1 mg dexamethasone under ultrasound guidance. The patient returned to rehabilitation and physical therapy in the school's training room, with improvement in symptoms with plans to return to compete in the spring season. A month later, he started training for his spring season and the symptoms returned. A repeat hydrodissection with a corticosteroid injection was tried again to get him through the season, but it failed.

### 2.2. Surgical Treatment

**Figure 4:**
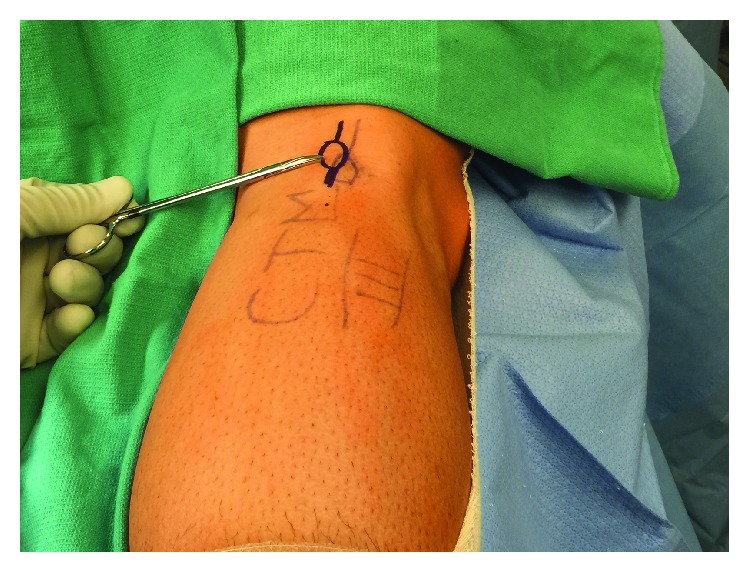
Preoperative marking of the fabella with the patient.

**Figure 5:**
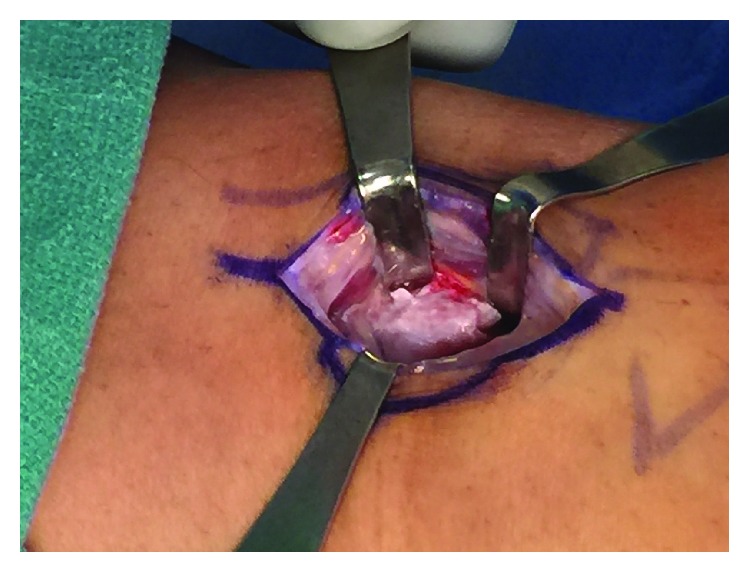
Exposure of the fabella.

**Figure 6:**
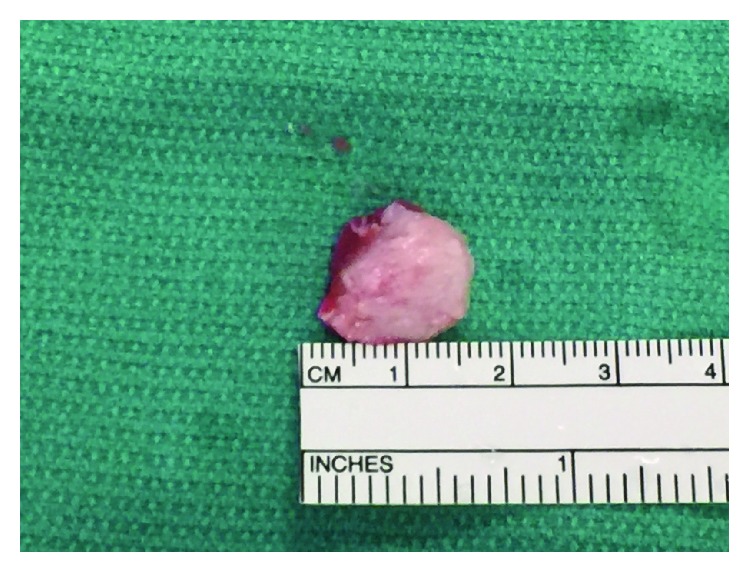
Fabella was removed as a single unit and measured 13 mm × 10 mm.

Preoperatively, we were able to palpate and mark the fabella with the patient (Figure 4). A tourniquet is used throughout the case to limit bleeding. We used a longitudinal incision directly over the fabella. The common peroneal nerve was identified, decompressed, and retracted laterally. The lateral gastrocnemius tendon was incised longitudinally directly over the fabella (Figure 5). The fabella itself was then removed as a single unit and measured 13 mm in length by 10 mm in width (Figure 6). There was an articulation of the fragment with the posterior femur, which was exposed. Great care is taken to protect the nerve while the exposure is closed in layers.

Postoperatively, the patient reported that the numbness had resolved from before surgery. He was kept touchdown weight bearing to allow the gastrocnemius tendon to heal. Physical therapy was started immediately after surgery to work on a knee range of motion. At 6 weeks, he was allowed to progress with a return to sport protocol and he returned symptom-free to track activities at 12 weeks. He remains asymptomatic over 2 years since his surgery.

## 3. Discussion

The exact function of the fabella continues to be unknown. The fabella is a sesamoid bone located in the lateral head of the gastrocnemius tendon [3]. It is usually deemed of minimal clinical significance, but it can cause clinically significant pathologies. The fabella is subject to acute and chronic problems such as fracture [19], osteoarthritis [20], popliteal artery entrapment [21], chondromalacia [22], dislocation [23], snapping after total knee arthroplasty [24], snapping in a native knee [4], and peroneal palsy [11].

There are many well-known causes of peroneal neuropathies including total knee arthroplasty [25], proximal tibial osteotomy [26], knee dislocation [27], and ganglion cyst [28]. There is far less in the literature about the fabella being a cause of peroneal neuropathy. In an anatomic study, Yamamoto and Ito found that 20.8% of common peroneal nerves pass through the surface of the fabella predisposing patients to peroneal neuropathy in hyperextension [3]. Sekiya et al. also found that when the common peroneal nerve passed posterior or lateral to the fabella, the nerve became wider and thinner than when it is located proximal to the fabella [29]. Park et al. noted that at the time of surgery, the peroneal nerve had a “sun-blushed” appearance and was slightly flattened at the level of the fabella [9].

Peroneal neuropathy caused by the fabella appears to have a bimodal distribution. As our patient demonstrated, there appears to be a group of patients in their 20's and 30's involved in athletics that developed peroneal neuropathy caused by the fabella. Tajima and Hotta reported on a 34-year-old volleyball player that required removal of the fabella and a 22-year-old ping-pong player that was treated with conservative measures that both went on to the resolution of symptoms [10]. Cesmebasi et al. report on a 22-year-old male runner who required an ultrasound-guided block and a lateral heel wedge that resolved his symptoms [17]. The second group usually involved patients in their 50's and 60's that developed symptoms through atraumatic causes. Cesmebasi et al. also reported on 3 patients aged 54, 56, and 56 who all developed symptoms from atraumatic causes [17]. Park et al. described a 67-year-old male who has 18 months of symptoms that were likely exacerbated by having polio since he was a child [9]. Tajima and Hotta also had a 53-year-old patient who developed symptoms after squatting that was able to be treated conservatively [10].

Ultrasound is a valuable tool not only in the diagnosis but also in the treatment on peroneal neuropathy caused by the fabella. While the X-ray is good for determining the bony fabella, 57.9% of the fabella composed of cartilage will not be seen on the X-ray [3]. Segal et al. were able to determine the ultrasound appearance of the fabella as hyperechoic with posterior acoustic shadowing [30]. Patel et al. described the use of a dynamic ultrasound to reproduce the snapping of the fabella over the femoral component of a total knee arthroplasty as the patient actively flexed and extended her knee [24]. Cesmebasi et al. reported on four patients that underwent dynamic ultrasound for diagnosis of peroneal neuropathy caused by the fabella [17]. They also performed an ultrasound-guided common peroneal block with local anesthetic for diagnostic and therapeutic purposes [17].

Treatment for a fabella causing peroneal neuropathy should be conservative, but surgical treatment should be reserved as a last option. First-line treatment is nonsteroidal anti-inflammatory medications with neuropathic pain-modifying agents as needed [17]. Physical therapy, bracing, and other modalities may be considered and have had a good result with fabella syndrome [31]. Tajima and Hotta report that 4 of their 7 patients were able to be treated conservatively and symptoms resolved in 1 to 2 months [10]. The next line of treatment is typically ultrasound-guided anesthetic block for diagnostic and therapeutic purposes. Cesmebasi et al. reported that they were able to treat 3 patients nonsurgically with an ultrasound-guided common peroneal nerve block, but 1 patient continued to have symptoms and required surgical excision [17]. Surgical treatment involves removal of the fabella with decompression of the nerve at the level of the fabella. Good results have been shown with resolution of the symptoms and even an increase in strength with motor deficits [9, 10, 17].

## 4. Conclusion

Patients with peroneal neuropathy caused by the fabella usually present with posterolateral knee pain that radiates anteriorly. Young athletes may be at risk for developing symptoms based on their sport. Patients will usually present after a long period of evaluation. First-line treatment is usually conservative with a combination of mediation, physical therapy, and bracing. Ultrasound is a valuable tool for dynamic evaluation and injection for diagnostic and therapeutic purposes. If nonoperative treatment does not resolve symptoms, good results may be obtained with surgical excision of the fabella and decompression of the peroneal nerve at the level of the fabella.
